# Diagnostic and Economic Evaluation of [^18^F]FDG PET/CT Versus MRI for Lymph Node Staging in Nasopharyngeal Carcinoma: Implications for Individualized Upper-Neck-Only Irradiation

**DOI:** 10.3390/jcm15051849

**Published:** 2026-02-28

**Authors:** Ya-Nan Zhao, Xiao-Wen Lan, Yun He, Xiao-Hui Wang, Chun-Yan Chen, Xu Zhang, Pu-Yun Ouyang, Fang-Yun Xie

**Affiliations:** 1 Department of Radiation Oncology, State Key Laboratory of Oncology in South China, Collaborative Innovation Center for Cancer Medicine, Guangdong Key Laboratory of Nasopharyngeal Carcinoma Diagnosis and Therapy, Guangdong Provincial Clinical Research Center for Cancer, Sun Yat-sen University Cancer Center, No. 651 Dongfeng Road East, Guangzhou 510060, China; zhaoyn@sysucc.org.cn (Y.-N.Z.);; 2 Department of Radiation Oncology, Sun Yat-sen Memorial Hospital, Sun Yat-sen University, Guangdong Provincial Key Laboratory of Malignant Tumor Epigenetics and Gene Regulation, Sun Yat-sen Memorial Hospital, Sun Yat-sen University, Guangzhou 510120, China; lanxw3@mail.sysu.edu.cn; 3 Department of Radiology, State Key Laboratory of Oncology in South China, Collaborative Innovation Center for Cancer Medicine, Guangdong Key Laboratory of Nasopharyngeal Carcinoma Diagnosis and Therapy; Guangdong Provincial Clinical Research Center for Cancer, Sun Yat-sen University Cancer Center, No. 651 Dongfeng Road East, Guangzhou 510060, China; 4 Department of Nuclear Medicine, State Key Laboratory of Oncology in South China, Collaborative Innovation Center for Cancer Medicine, Guangdong Key Laboratory of Nasopharyngeal Carcinoma Diagnosis and Therapy, Guangdong Provincial Clinical Research Center for Cancer, Sun Yat-sen University Cancer Center, No. 651 Dongfeng Road East, Guangzhou 510060, China

**Keywords:** nasopharyngeal carcinoma, MRI, PET/CT, N-staging, cost-effectiveness

## Abstract

**Background/Objectives:** To compare diagnostic performance and cost-effectiveness of [^18^F]FDG PET/CT versus MRI for cervical lymph node assessment in nasopharyngeal carcinoma (NPC) and to evaluate their impact on N-staging and upper-neck-only irradiation planning. **Materials and Methods:** We retrospectively identified treatment-naïve NPC patients who underwent both MRI and FDG PET/CT within 14 days prior to ultrasound-guided biopsy of specific cervical lymph nodes with rigorous one-to-one multimodal matching. Using histopathology as the reference standard (Cohort A, node level), sensitivity, specificity, positive predictive value (PPV), negative predictive value (NPV), and area under the curve (AUC) were compared between PET/CT and MRI. In a staging cohort (Cohort B, patient level), we compared imaging-based N-staging accuracy and the pathology-concordant classification of treatment recommendations assuming upper-neck-only irradiation for N0 to N1 disease. In discordant cases (Cohort C), three experienced radiation oncologists designed dose prescriptions and neck irradiation volumes, first using MRI alone and then after reviewing PET/CT to quantify decision impact. A decision tree/Markov model (10-year horizon) evaluated cost-effectiveness of MRI- versus PET/CT-initiated strategies. **Results:** In total, 694 biopsy-verified lymph nodes from 649 patients were analyzed. PET/CT demonstrated higher sensitivity (96.0% vs. 92.6%, *p* = 0.001) and NPV (80.2% vs. 66.7%, *p* < 0.001) than MRI, with comparable specificity (64.0% vs. 59.0%, *p* = 0.317) and PPV (91.4% vs. 90.0%, *p* = 0.203); AUCs were 0.864 and 0.841, respectively (*p* = 0.298). In Cohort B (N = 503), PET/CT provided accurate N-staging for a significantly higher proportion of patients compared to MRI (8.0% vs. 4.2%, *p* = 0.021) and yielded more accurate recommendations for upper-neck-only irradiation when restricted to N0 to N1 disease (93.8% vs. 88.9%, *p* = 0.003). In discordant cases (Cohort C, N = 62), PET/CT substantially improved accuracy compared with MRI and prompted clinically meaningful plan adjustments, including dose escalation for metastatic nodes (up to 16.7%) and expansion from upper-neck-only to whole-neck irradiation with rates of 6.4%, 8.0%, and 11.3% for the three radiation oncologists, respectively. In the base case economic analysis, PET/CT achieved higher effectiveness (5.329 vs. 5.305 quality-adjusted life years [QALYs]) at higher cost (US$27,228 vs. US$25,596), with an incremental cost–effectiveness ratio (ICER) of approximately US$68,000 per QALY, remaining below a willingness-to-pay threshold of US$100,000 per QALY; probabilistic sensitivity analysis favored PET/CT in 79.6% of iterations. **Conclusions:** FDG PET/CT provided superior sensitivity and negative predictive value versus MRI for detecting nodal metastases in NPC, improving pathology-adjudicated N-staging and the accuracy of upper-neck-only irradiation recommendations. PET/CT was cost-effective in the modeled setting, although treatment de-escalation for benign nodes remained conservative in clinical decision-making.

## 1. Introduction

The 2023 Chinese Society of Clinical Oncology (CSCO) Guidelines for the Diagnosis and Treatment of nasopharyngeal carcinoma, for the first time, recommend reducing the scope of neck radiotherapy for N0–1 patients from the entire neck to the upper neck [1]. This recommendation was based on evidence from a multicenter, randomized, phase III non-inferiority trial conducted by Tang et al., which demonstrated that elective upper-neck-only irradiation was non-inferior to whole-neck irradiation in terms of regional recurrence-free survival, while being associated with lower radiotherapy-related toxicity [2]. As lower-neck-sparing approaches increasingly diffuse into clinical practice, the identification of occult metastases becomes more critical than ever; failing to detect a positive node could lead to geographic misses and subsequent nodal relapses.

Magnetic resonance imaging (MRI) is currently the standard modality for NPC staging [3]. However, its reliance on morphological criteria—such as size and necrosis—limits its diagnostic accuracy, particularly for small or indeterminate lymph nodes. Notably, pathological validation studies in head-and-neck squamous cell carcinoma have reported MRI sensitivities as low as 60–76% [4,5], and in oral cavity carcinoma with palpably negative necks, sensitivity can drop to 22.2% [6] This diagnostic uncertainty is particularly challenging in NPC management. Unlike other head-and-neck malignancies, NPC is primarily treated with definitive radiotherapy, thereby lacking the definitive histopathological confirmation provided by surgical resection. Consequently, clinicians frequently encounter equivocal N-stages, creating a dilemma of potential undertreatment (recurrence risk) or overtreatment (unnecessary toxicity).

To address these limitations, [^18^F]fluorodeoxyglucose (FDG) positron emission tomography/computed tomography (PET/CT) is being utilized more frequently at initial diagnosis [7]. While PET/CT provides metabolic information that may complement anatomical imaging, substantial discrepancies remain between the two modalities. Reports indicate a concordance rate for N-stage of only 64.9% [8]. When MRI and PET/CT findings contradict each other, the lack of accessible pathological verification for multiple nodes often leaves the radiation oncologist in a dilemma regarding which modality to trust for target volume delineation. Furthermore, considering that PET/CT is significantly more resource-intensive than MRI, its routine application for N-staging requires rigorous economic justification alongside diagnostic validation.

Therefore, we conducted a study anchored by ultrasound-guided biopsy of specific lymph nodes to resolve these discrepancies. We hypothesized that [^18^F]FDG PET/CT would provide superior diagnostic performance compared to MRI in this pathology-verified cohort, thereby improving N-staging accuracy and enabling cost-effective decision-making for upper-neck-only irradiation.

## 2. Materials and Methods

### 2.1. Study Design and Patients

This retrospective study was approved by the Ethics Committee of Sun Yat-sen University Cancer Center (SYSUCC) (No. B2021-059-01; No. B2023-012-01); informed consent was waived due to the retrospective design. From January 2015 to August 2021, we screened pathology reports to identify treatment-naïve NPC patients who underwent ultrasound-guided nodal biopsy within 14 days (2 weeks) after completing both MRI and [^18^F]FDG PET/CT. To ensure precise one-to-one correspondence, each biopsied node was rigorously matched across modalities by an experienced radiation oncologist (Pu-Yun OuYang [P.Y.O.], with over ten years of experience in NPC). A lymph node was included only if the biopsied target on ultrasound shared identical size, location, capsule integrity, and anatomic relationship with surrounding tissues as the specific node identified on MRI and PET/CT images. The identified nodes were contoured using ITK-SNAP version 3.6.0 and assigned to appropriate levels according to consensus guidelines [9].

For clarity, “nodal levels” indicate anatomic compartments for localization/contouring according to consensus guidelines [10], whereas “N-stage” denotes TNM staging categories (N0 to N3) [11,12] determined at the patient level based on the distribution (laterality and regions/levels) and size criteria of involved nodes.

### 2.2. Cohort Definitions

Three nested cohorts were defined to address diagnostic, staging, and clinical decision-making objectives.

Cohort A (diagnostic; node level): This cohort included biopsy-verified lymph nodes from treatment-naïve NPC patients who underwent both MRI and [^18^F]FDG PET/CT before any therapy. This cohort consisted exclusively of nodes that satisfied the stringent inclusion criteria for one-to-one multimodal matching described above. It served to compare the diagnostic performance (sensitivity, specificity, predictive values, and receiver operating characteristic (ROC) performance) of PET/CT versus MRI using histopathology as the reference standard.

Cohort B (staging; patient level): A subset of patients from Cohort A, used to evaluate the accuracy of N-staging. Patients were included if their PET/CT- and MRI-based N-stages were concordant; if discordant, the ultrasound-guided biopsy of the relevant lymph node served as the reference standard to adjudicate which imaging modality provided the correct N-stage. It was used to evaluate the accuracy of imaging-based N-staging and the appropriateness of upper-neck-only irradiation recommendations derived from each modality.

Cohort C (decision; clinical practice level): This subset consisted of patients from Cohort A with discordant MRI and PET/CT findings. Three experienced radiation oncologists (XWL, XHW, and CYC), each with more than 10 years of clinical experience in NPC, independently designed dose prescriptions and irradiation volumes, first using MRI data alone and, subsequently, after reviewing PET/CT results. This cohort was used to quantify the clinical impact of PET/CT on treatment planning decisions. Cohort C was designed to quantify decision impact (dose category and field extent) and did not involve full contouring on a simulation CT dataset.

### 2.3. Imaging Acquisition and Interpretation

MRI and PET/CT scans were performed within two weeks prior to biopsy following institutional protocols (acquisition parameters detailed in Supplementary Methods) [13]. To ensure rigorous node-to-node verification, the specific regions of interest (ROIs) corresponding to the biopsied nodes in Cohort A were presented to expert radiologists for targeted evaluation. Images were reviewed independently by two nuclear medicine specialists (including author XZ, >20 years’ experience) and two head-and-neck MRI radiologists (including author YH, interpreting > 300 NPC cases/month). All readers were strictly blinded to clinical information and pathological results. PET/CT acquisition and reconstruction were performed on integrated systems (Discovery ST or Biograph mCT) using institutional protocols consistent with published guidelines, without major protocol changes during the study period; scanner-specific parameters are provided in the Supplementary Methods.

Nodal integrity was assessed using a 5-point confidence scale: 0 (definitely benign), 1 (low suspicion), 2 (moderate suspicion), 3 (high suspicion), and 4 (definitely metastatic). To prioritize the detection of occult metastases and minimize false negatives, a conservative threshold was applied: scores of 2 to 4 were classified as node-positive for statistical analysis (cutoff ≥ 2).

Diagnostic criteria for malignancy were standardized according to established consensus guidelines [5,14,15,16,17]. Morphologically, lymph nodes were considered positive on MRI or CT if they exhibited a short-axis diameter >10 mm (or >5 mm for retropharyngeal nodes), necrosis, irregular margins, loss of fatty hilum, extracapsular spread, or varying degrees of asymmetry. Metabolically, PET positivity was defined as visually prominent [^18^F]FDG uptake exceeding background blood pool activity that could not be attributed to physiologic biodistribution. Semi-quantitative measures (e.g., SUVmax and lesion-to-blood pool uptake ratios) were available on the workstation and were used as supportive information when assigning the five-point confidence score; however, no fixed SUV cutoff was mandated due to potential variability across scanners and reconstruction settings in this retrospective cohort. For the final PET/CT classification, radiologists performed a holistic assessment, integrating metabolic intensity with anatomical localization on the intrinsically co-registered PET/CT fused images (PET overlay on the CT acquired in the same session).

### 2.4. Radiation Oncologist Decision Assessment (Cohort C)

Three radiation oncologists independently assigned a dose category—low (<60 Gy), intermediate (60–66 Gy), or radical (≥66 Gy)—and a neck irradiation volume (upper-neck-only vs. whole-neck) for each patient. Assessments were performed in two phases: first using MRI data alone and, subsequently, after reviewing PET/CT images.

### 2.5. Statistical Analysis

Sensitivity, specificity, positive predictive value (PPV), and negative predictive value (NPV) were compared using the McNemar test [18] and weighted generalized score statistics [19] (R package DTComPair, v4.3.1). The area under the receiver operating characteristic curve (AUC) was compared using the DeLong test. Differences in N-stage distribution and the appropriateness of upper-neck-only irradiation plans were evaluated using the paired McNemar test. A two-sided *p* < 0.05 was considered statistically significant.

### 2.6. Cost–Effectiveness Model

A decision tree and Markov state-transition model (1-year cycle; 10-year horizon) was constructed using TreeAge Pro 2022 (R1)(TreeAge Software, Williamstown, MA, USA) to compare diagnostic strategies initiating with MRI versus PET/CT. Transition probabilities were parameterized using the diagnostic accuracy estimates observed in Cohort A and contemporary literature (Supplementary Table S1; Supplementary Figure S1). Costs and health utilities were expressed as US$ per quality-adjusted life year (QALY), adopting a willingness-to-pay (WTP) threshold of US$100,000/QALY.

Key Model Assumption: Informed by regional recurrence patterns [20], we conservatively assumed that metastatic lymph nodes misclassified as benign (false negatives)—and subsequently undertreated with upper-neck-only irradiation—would carry a regional recurrence risk exceeding 25% [20,21,22].

To evaluate model robustness, both deterministic (tornado) and probabilistic sensitivity analyses (Monte Carlo simulations with 1000 iterations) were performed. Detailed model structure and input parameters are provided in the Supplementary Methods.

## 3. Results

### 3.1. Patients

**Figure 1:**
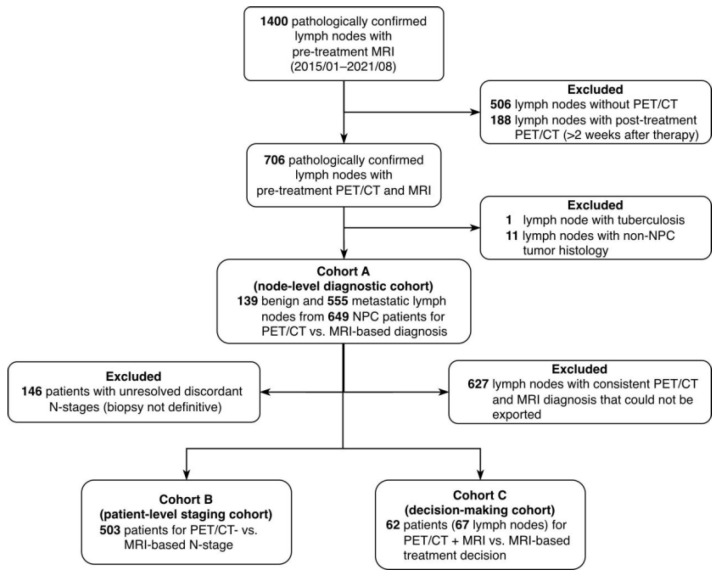
Patient inclusion flow and cohort classification for diagnostic, staging, and decision-making analyses. Abbreviations: MRI, magnetic resonance imaging; PET/CT, [^18^F]FDG positron emission tomography/computed tomography; NPC, nasopharyngeal carcinoma.

As shown in Figure 1, Cohort A comprised 694 lymph nodes from 649 patients and was used to compare the diagnostic reliability of PET/CT and MRI as interpreted by expert radiologists. A representative lymph node is illustrated in Supplementary Figure S2. Cohort B included 503 patients and was used to evaluate the accuracy of N-staging and the appropriateness of upper-neck-only irradiation plans derived from PET/CT and MRI, respectively. Cohort C consisted of 67 lymph nodes from 62 patients and was designed to assess the impact of imaging modality on radiotherapy decision-making, including dose prescription and neck irradiation volumes determined by experienced radiation oncologists. Baseline patient and nodal characteristics are summarized in Table 1.

### 3.2. Diagnostic Performance (Cohort A; 694 Nodes)

**Figure 2:**
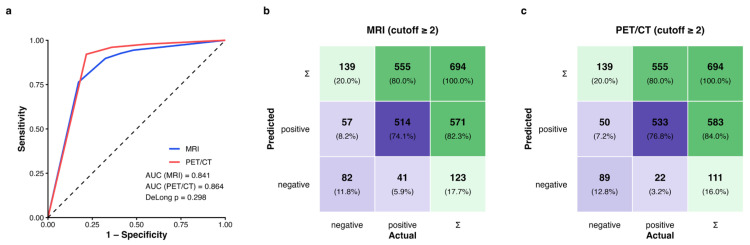
Diagnostic performance of MRI and PET/CT for metastatic cervical lymph nodes in Cohort A (n = 694). (**a**) ROC curves with corresponding AUC. (**b**,**c**) confusion matrix heatmaps illustrating the distributions of TP, TN, FP and FN for MRI and PET/CT, respectively, using histopathology as the reference standard. Abbreviations: MRI, magnetic resonance imaging; PET/CT, [^18^F] fluorodeoxyglucose positron emission tomography/computed tomography; ROC, receiver operating characteristic; AUC, area under the curve; TP, true positive; TN, true negative; FP, false positive; FN, false negative.

PET/CT correctly classified 622/694 nodes (89.6% [95% CI, 87.1–91.7%]) vs. 596/694 (85.9% [83.1–88.3%]) for MRI. PET/CT yielded higher sensitivity (96.0% [94.1–97.4%] vs. 92.6% [90.1–94.5%], *p* = 0.001) and NPV (80.2% [71.8–86.5%] vs. 66.7% [57.9–74.4%], *p* < 0.001) with comparable specificity (64.0% [55.8–71.5%] vs. 59.0% [50.7–66.8%], *p* = 0.317) and PPV (91.4% [88.9–93.4%] vs. 90.0% [87.3–92.2%], *p* = 0.203) (Table 2). AUCs were 0.864 (PET/CT) and 0.841 (MRI), DeLong *p* = 0.298 (Figure 2).

### 3.3. Effect on N-Staging and Upper-Neck-Only Recommendation (Cohort B; 503 Patients)

**Figure 3:**
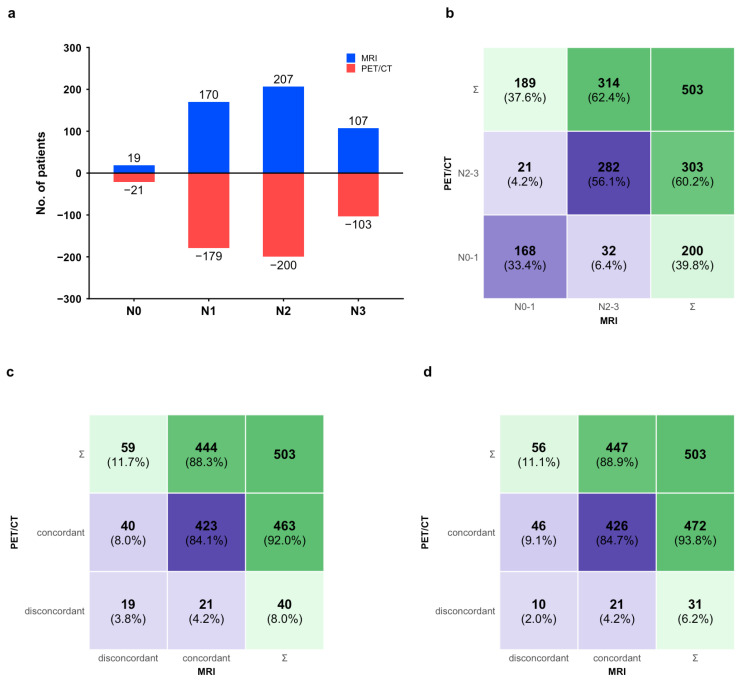
Patient-level impact of MRI versus [^18^F]FDG PET/CT on N-staging and treatment decision-making (N = 503). (**a**) Distribution of clinical N-stage assigned by MRI and PET/CT. (**b**–**d**) Confusion matrix heatmaps: (**b**) agreement in binned N-stage (N0–1 vs. N2–3) between PET/CT and MRI; (**c**) diagnostic consistency of PET/CT and MRI; (**d**) consistency of treatment recommendations assuming selective upper-neck-only irradiation for N0–1 disease, comparing recommendations for upper-neck-only versus whole-neck irradiation by PET/CT and MRI.

PET/CT assigned slightly more N0 to N1 stages than MRI (N0: 4.2% [21/503] vs. 3.8% [19/503]; N1: 35.6% [179/503] vs. 33.8% [170/503]) (Figure 3a). However, the overall distribution of N0–1 versus N2–3 did not differ significantly between modalities (*p* = 0.170) (Figure 3b). With biopsy adjudication, PET/CT correctly staged more patients (8.0% [40/503]) than MRI (4.2% [21/503], *p* = 0.021); both modalities were concordantly correct in 84.1% (423/503) and concordantly incorrect in 3.8% (19/503) (Figure 3c). Assuming eligibility for upper-neck-only irradiation was restricted to N0 to N1, PET/CT-guided recommendations were correct in 93.8% (472/503) versus 88.9% (447/503) for MRI (*p* = 0.003) (Figure 3d).

### 3.4. Influence on Dose and Irradiation Fields Recommendations (Cohort C; 62 Patients/67 Nodes)

In this cohort of discordant cases, the diagnostic superiority of PET/CT over MRI was evident. MRI radiologists correctly identified only 16.7% (4/24) of pathologically metastatic nodes and 41.9% (18/43) of benign nodes. In contrast, PET/CT radiologists achieved correctness rates of 83.3% (20/24) and 58.1% (25/43), respectively.

**Figure 4:**
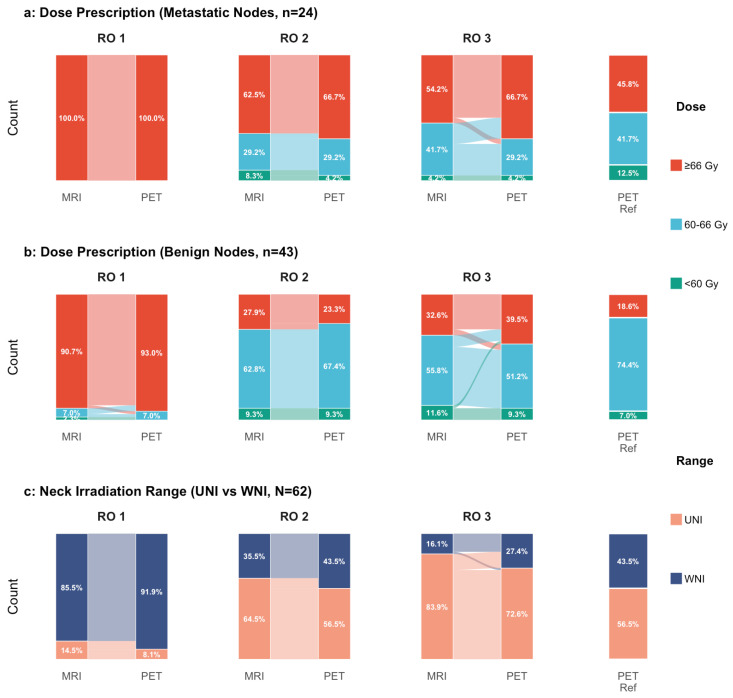
Sankey diagrams illustrating changes in radiotherapy dose recommendations and neck irradiation fields before and after PET/CT review by three radiation oncologists (RO1 to RO3) in Cohort C. (**a**) Changes in dose recommendations for metastatic lymph nodes. (**b**) Changes in dose recommendations for benign lymph nodes. (**c**) Changes in irradiation-field recommendations between selective upper-neck-only irradiation and whole-neck irradiation. Abbreviations: MRI, magnetic resonance imaging; PET/CT, [^18^F] fluorodeoxyglucose positron emission tomography/computed tomography; RO, radiation oncologist.

Reflecting the diagnostic uncertainty of MRI, radiation oncologists’ decisions varied significantly and often deviated from the pathological ground truth. Without PET/CT information, 37.5% (9/24) and 45.9% (11/24) of the metastatic lymph nodes were designated for sub-radical doses (<66 Gy) by two radiation oncologists, posing a risk of undertreatment (Figure 4a). Conversely, 90.7% (39/43), 27.9% (12/43), and 32.6% (14/43) of benign lymph nodes were heavily overtreated (Figure 4b). Recommendations for upper-neck-only irradiation varied widely among the three oncologists (ranging from 14.5% to 83.9%), highlighting the lack of consensus when relying solely on MRI (Figure 4c).

PET/CT results prompted clinically meaningful adjustments in treatment planning. For metastatic nodes, oncologists escalated the prescribed dose in up to 16.7% (4/24) of cases, potentially salvaging patients from under-dosing (Figure 4a). For benign nodes, the integration of PET/CT resulted in bidirectional dose adjustments. While PET/CT facilitated dose de-escalation in 4.7% (2/43) of cases consistently across all three oncologists, it also prompted dose escalation in 0% to 11.6% (0/43 to 5/43) of benign nodes (Oncologist 1: 7.0%; Oncologist 2: 0%; Oncologist 3: 11.6%), reflecting the influence of false-positive PET uptake (Figure 4b). Regarding target volume, the three oncologists consistently expanded treatment plans from upper-neck-only to whole-neck irradiation after identifying occult lower-neck involvement on PET/CT, with rates of 6.4%, 8.0%, and 11.3% for Oncologists 1 to 3, respectively (Figure 4c).

### 3.5. Cost–Effectiveness

**Figure 5:**
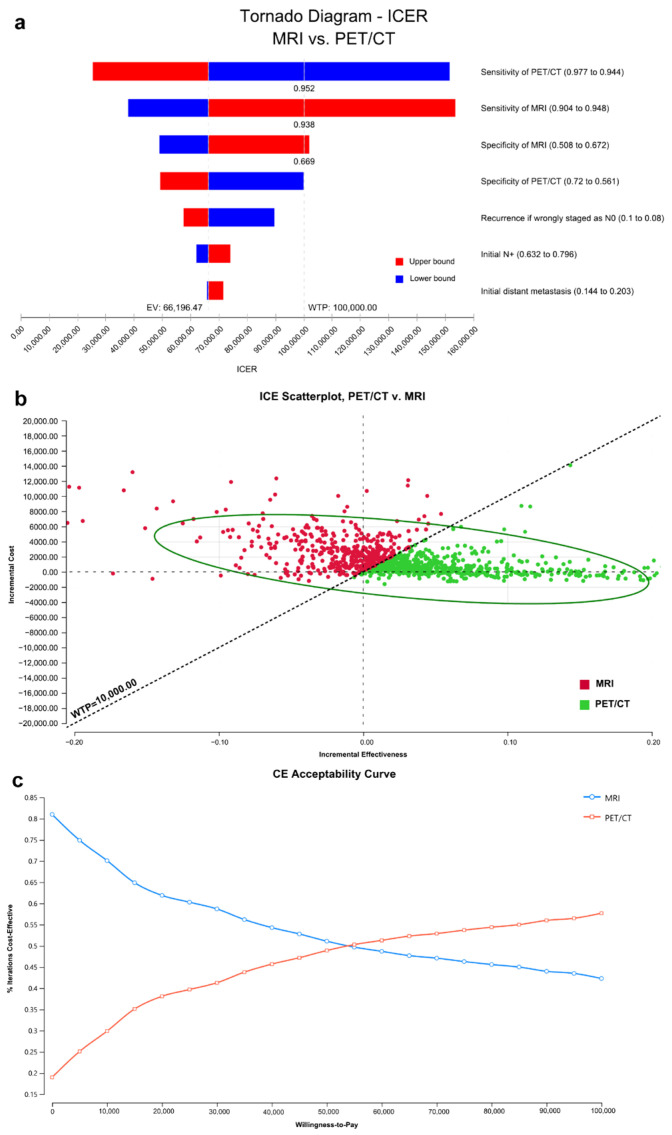
The tornado diagram of the deterministic sensitivity analysis (**a**), scatterplot of Monte Carlo simulation. Red bars indicate the ICER when the parameter is set at its upper bound, and blue bars indicate the ICER when the parameter is set at its lower bound (relative to the base-case value). (**b**), and cost-effectiveness acceptability curve (**c**) bars in the tornado diagram indicated the impact of variation in input parameters on incremental cost–effectiveness ratio starting from the expected value in the base case scenario for comparing PET/CT with MRI. Abbreviations: ICER, incremental cost–effectiveness ratio; MRI, magnetic resonance imaging; PET/CT, [^18^F] fluorodeoxyglucose positron emission tomography/computed tomography; WTP, willingness to pay.

Base case analysis showed that PET/CT yielded higher effectiveness (5.329 vs. 5.305 QALYs) at a higher cost (US$27,228 vs. US$25,596) compared with MRI. The Incremental cost–effectiveness ratio (ICER) was approximately US$68,000/QALY, which remained below the US$100,000/QALY willingness-to-pay (WTP) threshold. In deterministic sensitivity analysis, PET/CT remained the preferred strategy across most parameter ranges but ceased to be cost-effective (ICER > WTP) if PET/CT sensitivity fell below 95.2% or if MRI sensitivity exceeded 93.8% with specificity >66.9%. Probabilistic sensitivity analysis demonstrated that PET/CT was cost-effective in 79.6% of iterations (Figure 5).

One-way sensitivity analysis demonstrated that the assumed prevalence of nodal metastasis influenced the ICER, but its impact was smaller than that of diagnostic sensitivity parameters (Figure 5a). Across the tested prevalence range (0.632–0.796), the ICER remained below the willingness-to-pay threshold.

## 4. Discussion

Based on pathological confirmation and within-lymph nodes comparison, we demonstrated the greater sensitivity and negative predictive value of PET/CT than MRI in diagnosing and staging the lymph nodes of nasopharyngeal carcinoma. PET/CT improved the accuracy of N-staging compared with MRI, thereby facilitating the selection of more patients for upper-neck-only irradiation. Still, some lymph nodes were overestimated as metastatic and consequently tended to receive radical radiotherapy doses. They changed the dose prescriptions and neck irradiation plans in some instances after being informed of the PET/CT results, but the changes were minor. For better upper-neck-only irradiation, PET/CT was warranted and cost-effective to diagnose and stage lymph nodes correctly.

Because our primary definition of positivity (scores 2–4 as positive; cutoff ≥ 2) intentionally prioritized sensitivity, it may increase false-positive findings and potential overtreatment. To evaluate the robustness of our conclusions, we performed a secondary analysis using a stricter threshold (scores 3–4 as positive; cutoff ≥ 3; Supplementary Table S2). As expected, specificity improved for both modalities with only a modest decrease in sensitivity, and PET/CT continued to outperform MRI at cutoff ≥ 3. These findings indicate that the observed advantage of PET/CT is not driven by a single threshold choice. Clinically, a stricter threshold could mitigate unnecessary treatment escalation, whereas a lower threshold may be preferred when tolerance for false negatives is low, particularly in the context of selective upper-neck irradiation.

As reported in the previous study [23], patients with nasopharyngeal carcinoma were most likely to have enlarged lymph nodes in levels II and III. Because performing fine-needle aspiration of retropharyngeal lymph nodes is risky, it was unexpected that the proportion of lymph nodes in level VII was much smaller herein than in the previous reports [9,23]. Notably, level VIII lymph nodes were only second to level II in the percentage of biopsied lymph nodes. Given that the likelihood of parotid lymph node metastasis in nasopharyngeal carcinoma is as low as 2.5% [24], pathologic diagnosis of the enlarged lymph nodes here is, thus, highly desired to determine whether the ipsilateral level VIII should be contoured in the low-risk clinical target volume of radiotherapy. Although the inclusion criteria of our study were established based on the biopsied lymph nodes, the distribution of primary T-stages in the final included patients was highly comparable to that of the clinical trial [2] investigating the safety of sparing lower-neck irradiation to N0–N1 patients.

Our finding that PET/CT was more sensitive than MRI was similarly observed by a prior study [25], in which metastatic neck lymph nodes were diagnosed by imaging follow-up instead of histopathology. In addition, a previous study [26] also found the superiority of PET/CT over MRI in diagnosing the recurrent lymph nodes of nasopharyngeal carcinoma after chemoradiotherapy. The differences were that PET/CT demonstrated higher specificity than MRI in distinguishing benign and recurrent lymph nodes [26], whereas PET/CT performed better than MRI in diagnosing untreated lymph nodes with higher sensitivity herein. Both PET/CT and MRI were more sensitive and less specific in diagnosing treatment naïve lymph nodes than in diagnosing lymph node recurrence after chemoradiotherapy. However, similar to the more accurate PET/CT-based rN-stage resulting from its higher specificity in diagnosing recurrent lymph nodes [26], its higher sensitivity in diagnosing untreated lymph nodes ultimately led to the more accurate PET/CT-based N-stage. In a prior study [27], MRI-staged T3N0-3M0 patients had nondifferent survival rates as they were the same T3N1M0 if staged by PET/CT, which indirectly indicated that PET/CT was possibly more accurate than MRI in N-stage. Via biopsying the key lymph nodes that caused the discrepancies between PET/CT-based and MRI-based N-stages in the present study, we firmly validated again that PET/CT was more likely correct. Although the absence of neck dissection possibly omitted occult metastatic lymph nodes, our study showed the most convincing comparison results.

The questionnaire showed that MRI-based dose prescriptions and neck irradiation plans by different radiation oncologists varied greatly. Without knowing the diagnostic advantages of PET/CT, radiation oncologists correctly identified more metastatic lymph nodes with radical radiation doses and corrected some inappropriate upper-neck-only irradiation plans after referring to the PET/CT diagnosis. But they still did not dare to lower the irradiation doses to the lymph nodes that were proven benign if biopsied. Considering that the negative predictive value of PET/CT was only 80.2% in cohort B and 86.2% in cohort C, it is still risky to de-escalate the dose to lymph nodes just based on the diagnosis of PET/CT. However, similar to the reliability in head-and-neck squamous cell carcinoma based on neck dissection [28,29], the negative predictive value of PET/CT was over 80% and was significantly much higher than that of MRI. Together with the higher sensitivity, PET/CT would undoubtedly assist in reducing the unqualified participants in multicenter clinical trials whose main inclusion criteria were T-stage and N-stage. In addition, PET/CT could also help radiation oncologists in dose prescriptions of lymph nodes and identifying suitable patients to receive upper-neck prophylactic irradiation, especially when diagnoses and N-stages by different modalities contradict each other. These findings suggest that PET/CT may facilitate workflow standardization through structured reporting and predefined management rules for discordant nodes, especially when upper-neck-only irradiation is being considered.

PET/CT has been recommended for nasopharyngeal carcinoma patients to detect potential distant metastasis at the initial diagnosis [30], especially for those at high risk of distant metastasis [31]. These patients only accounted for 10% [31]. However, suppose PET/CT is recommended to assist in diagnosing lymph nodes and N-stage. In that case, we have to calculate its cost-effectiveness because the pre-test probability of cervical nodal metastasis is 62.2–79.6% [32,33,34], but only 40% (Figure 3b) are ultimately staged with N0–1 by PET/CT and potentially eligible for upper-neck irradiation. The incremental cost–effectiveness ratio (ICER) was positive yet remained below the willingness-to-pay threshold, indicating that the PET/CT-initiated strategy achieved greater effectiveness at an acceptable additional cost. In Cohort A, the lymph nodes selected for ultrasound-guided biopsy were not subject to strict qualifying criteria and represented targeted biopsy candidates in routine practice. As a result, the diagnostic spectrum may have differed from that of an unselected node population. Moreover, because Cohort A consisted of ultrasound-guided biopsied target nodes with rigorous one-to-one multimodal matching, the prevalence of metastatic nodes was enriched (80% of biopsied nodes were metastatic), exceeding that reported in more general NPC cohorts [32,33,34]. This enrichment improves the precision of node-to-node pathology correlation, and it also introduces selection (spectrum/verification) bias and limits generalizability to broader screening settings. However, the cost–effectiveness model did not use the enriched 80% node-level prevalence in Cohort A; instead, it adopted an external pre-test probability of cervical nodal metastasis (baseline 69.1%, range 0.632–0.796; Supplementary Table S1). Importantly, PPV and NPV are prevalence-dependent. Under a lower and more representative nodal prevalence, PPV would be expected to decrease whereas NPV would increase. Therefore, the NPV observed in Cohort A should be interpreted cautiously as it may underestimate the rule-out performance of PET/CT and MRI in routine populations with lower metastatic-node prevalence. This may have contributed to increased sensitivity and positive predictive value. As a result, our MRI sensitivity exceeded prior findings [25,27]. The previous study [35] found that 19 of the 37 MRI-negative cervical lymph nodes were positive on PET/CT, and metastasis was confirmed by ultrasonography-guided fine-needle aspiration cytology in 16, which showed a sensitivity of 94.1% for PET/CT. So, the superiority of PET/CT over MRI in sensitivity was possibly underestimated despite its significance. Accordingly, the cost-effectiveness of PET/CT was likely to be more significant in the clinic, considering the high incidence of MRI-negative lymph nodes [31] or small lymph nodes [36] in nasopharyngeal carcinoma. In addition, our findings concurred with other reports that showed the cost-effectiveness of the initially more cost-intensive PET/CT in different oncologic diseases [37,38]. In one-way sensitivity analysis (Figure 5a), varying the pre-test probability within this reported range had a smaller impact on the ICER than diagnostic sensitivities, and the ICER remained below the willingness-to-pay threshold.

Because the ICER is sensitive to the assumed risk of regional recurrence when metastatic nodes are missed and, consequently, not covered by elective irradiation, we parameterized this transition probability conservatively. Specifically, we assumed a 3-year recurrence probability of 25.44% for metastatic nodes misclassified as N0 and managed with upper-neck-only irradiation (Supplementary Table S1), derived from the proportion of lower-neck involvement and the baseline regional relapse risk reported in the randomized trial that informed our de-escalation context [2,4]. Historical patterns-of-failure series also report substantial cervical nodal failure when elective neck irradiation is omitted, supporting the clinical plausibility of a meaningful recurrence risk if occult disease is undertreated [20,21]. This parameter was tested over a wide plausible range in deterministic and probabilistic sensitivity analyses (Figure 5a–c).

Importantly, despite the improved diagnostic performance of PET/CT, no prominent dose de-escalation was observed for pathologically benign lymph nodes in Cohort C. This reflects a conservative tendency in real-world clinical practice rather than a lack of diagnostic confidence. In the absence of prospective outcome data directly linking PET/CT-based nodal classification to long-term regional control, radiation oncologists remain reluctant to substantially reduce dose or omit coverage for nodes that appear metabolically inactive but are morphologically suspicious or clinically equivocal.

Such caution is understandable, as erroneous de-escalation carries a potentially irreversible risk of nodal recurrence; treatment intensification is generally perceived as more acceptable in the short term. Therefore, the observed conservative decision-making in Cohort C should not be interpreted as a failure of PET/CT to inform treatment planning but rather as a manifestation of the current evidence gap between improved diagnostic accuracy and outcome-validated de-escalation strategies. Future studies incorporating real-world PET/CT-based nodal staging with corresponding radiotherapy strategies and longitudinal oncologic outcomes are needed to determine whether current treatment intensity exceeds what is clinically necessary and to define safe thresholds for dose and volume reduction.

The major limitation was the insufficient representation of levels IV and Vb in the biopsied lymph nodes, which hindered direct evidence regarding the risk posed by sparing lower-neck irradiation. As the recent randomized controlled trial [2] concluded that as long as there were no metastatic lymph nodes on one side of the whole neck, irradiation to the lower neck on that side could be omitted, it is warranted and appropriate to focus on the diagnosis of all the enlarged lymph nodes. The chance of enlarged lymph nodes in the lower neck was minimal. However, lymph nodes in the upper neck often showed significant inflammation and enlargement. Thus, the upper-neck lymph nodes held particular importance, and their diagnostic results by PET/CT or MRI were adequate to inform whole-neck or upper-neck irradiation. In addition, it should be noted that selective biopsy was performed on patients, and due to the retrospective design of the study, the initial biopsy intentions could not be traced. This may have caused a large selection bias to arise, leading to a potential overestimation of the reliability, particularly the sensitivity, of PET/CT and MRI, which accounted for a higher prevalence of positive lymph nodes than has been historically reported [32,33,34]. However, the sensitivity superiority of PET/CT was evident in these easily diagnosable samples, and thus its reliability may be more apparent in clinical practice. Secondly, neither inter-observer agreement nor intra-observer repeatability was formally assessed (e.g., κ statistics or repeat reading after a washout period) because final classifications were reached by consensus; therefore, the reproducibility of image interpretation could not be quantified. Thirdly, we adopted a more cautious approach to defining the criteria for PET positivity based on prior research on nasopharyngeal carcinoma [39] and head-and-neck cancer [19,20]. As a result, we obtained a lower specificity, although morphological characteristics on CT scans were taken into account. However, we only did this as missing positive lymph nodes, underestimating N-stage, reducing radiotherapy dose incorrectly, and expanding the population for upper-neck irradiation erroneously raised the incidence of nodal recurrence, with consequences that were more intolerable than the side effects of prophylactic irradiation. Finally, we did not analyze the differences in PET/CT and MRI devices as we aimed to replicate clinical practice. Similarly, we did not include advanced MRI sequences, such as Diffusion-Weighted Imaging/Apparent Diffusion Coefficient, because they were not available in most cases.

Although we demonstrated that PET/CT alters N-staging and radiotherapy decision-making, we did not directly evaluate whether these changes improved regional control, toxicity, or patient-reported outcomes. A prospective study (or registry) that links PET/CT-informed nodal management to longitudinal outcomes is warranted, particularly in the era of elective upper-neck irradiation. Such a study could predefine management rules for discordant nodes and evaluate both effectiveness and consistency across clinicians.

## 5. Conclusions

[^18^F]FDG PET/CT demonstrates superior sensitivity and negative predictive value compared to MRI for nodal staging in NPC, particularly for distinguishing metastatic from benign lymph nodes in the upper neck. While clinical implementation remains cautious regarding dose de-escalation, the integration of PET/CT significantly improves the accuracy of N-staging and the appropriateness of upper-neck-only irradiation recommendations. From a health-economic perspective, PET/CT represents a cost-effective strategy for optimizing curative radiotherapy planning.

## Figures and Tables

**Table 1:** Demographic data of nasopharyngeal carcinoma patients.

	Cohort A	Cohort B	Cohort C
**No. of lymph nodes**	n = 694	n = 503	n = 67
**Metastatic**	555 (80.0%)	409 (81.3%)	24 (35.8%)
**Benign**	139 (20.0%)	94 (18.7%)	43 (64.2%)
**Metastatic lymph nodes levels**	n = 555	n = 409	n = 24
I	13 (2.3%)	5 (1.2%)	0
II	474 (85.4%)	351 (85.8%)	15 (62.5%)
III	22 (4.0%)	14 (3.4%)	2 (8.3%)
IV	4 (0.7%)	4 (1.0%)	0
Va	17 (3.1%)	16 (3.9%)	3 (12.5%)
Vb	4 (0.7%)	4 (1.0%)	1 (4.2%)
VII	2 (0.4%)	1 (0.2%)	0
VIII	19 (3.4%)	14 (3.4%)	3 (12.5%)
**Benign lymph nodes levels**	n = 139	n = 94	n = 43
I	11 (7.9%)	5 (5.3%)	1 (2.3%)
II	92 (66.2%)	70 (74.5%)	35 (81.4%)
III	7 (5.0%)	4 (4.3%)	2 (4.7%)
IV	4 (2.9%)	2 (2.1%)	0
Va	1 (0.7%)	0	0
Vb	0	0	0
VII	0	0	0
VIII	24 (17.3%)	13 (13.8%)	5 (11.6%)
**No. of patients**	N = 649	N = 503	N = 62
**Median age (IQR)**	45 (36.5–53)	46 (37–53)	44 (36–53.25)
**Male (%)**	482 (74.2)	374 (74.3)	49 (79.0)
**MRI-based T-stage**			
T1	84 (12.9%)	62 (12.3%)	9 (14.5%)
T2	64 (9.9%)	55 (10.9%)	4 (6.5%)
T3	358 (55.2%)	277 (55.1%)	30 (48.4%)
T4	143 (22.0%)	109 (21.7%)	19 (30.6%)

**Table 2:** Diagnostic performance of PET/CT vs. MRI at node level (cutoff ≥ 2).

Metric	PET/CT	MRI	*p* Value
ACC (95% CI)	89.6% (87.1–91.7%)	85.9% (83.1–88.3%)	-
SEN (95% CI)	96.0% (94.1–97.4%)	92.6% (90.1–94.5%)	0.001
SPE (95% CI)	64.0% (55.8–71.5%)	59.0% (50.7–66.8%)	0.317
PPV (95% CI)	91.4% (88.9–93.4%)	90.0% (87.3–92.2%)	0.203
NPV (95% CI)	80.2% (71.8–86.5%)	66.7% (57.9–74.4%)	<0.001
AUC	0.864	0.841	0.298

Abbreviations: ACC: accuracy; SEN: sensitivity; SPE: specificity; PPV: positive predictive value; NPV: negative predictive value; AUC: area under the curve.

## Data Availability

De-identified data supporting the findings of this study are available from the corresponding author upon reasonable request and with institutional approvals. Key study inputs and methodological details are provided in the Supplementary Materials.

## Supplementary Materials

The following supporting information can be downloaded at https://www.mdpi.com/article/10.3390/jcm15051849/s1, Figure S1: The economic decision tree (a) and Markov transition state model (b). MRI, magnetic resonance imaging; PET/CT, [18F] fluorodeoxyglucose positron emission tomography/computed tomography; N0, no cervical lymph node metastasis; N+, cervical lymph node metastasis; Figure S2: A pathological positive lymph node in level II of the left neck. It was correctly diagnosed by [18F] fluorodeoxyglucose positron emission tomography/computed tomography (a,b) but misdiagnosed by magnetic resonance imaging with sequences of T2-weighted imaging (c) and T1-weighted imaging with contrast (d); Table S1: Markov model input parameters; Table S2: Diagnostic performance of PET/CT vs. MRI at node-level (cutoff ≥ 3).

## Abbreviations

The following abbreviations are used in this manuscript:

NPC	nasopharyngeal carcinoma
CI	confidence interval
FSE-T1WI	fast spin–echo T1-weighted imaging
MRI	magnetic resonance imaging
PET/CT	[^18^F]FDG positron emission tomography/computed tomography
IMRT	intensity-modulated radiotherapy
ROC	receiver operating characteristic
AUC	area under the curve
PPV	positive predictive value
NPV	negative predictive value
QALY	quality-adjusted life year
ICER	incremental cost–effectiveness ratio
WTP	willingness-to-pay
