# Supplementary material for: Diagnostic and Economic Evaluation of [18F]FDG PET/CT Versus MRI for Lymph Node Staging in Nasopharyngeal Carcinoma: Implications for Individualized Upper-Neck-Only Irradiation
Source: J Clin Med. 2026 Feb 28;15(5):1849. doi: 10.3390/jcm15051849 (PMC12985516; doi:10.3390/jcm15051849)
Supplement: Supplementary file 1 [file jcm-15-01849-s001.zip › jcm-4126162-supplementary.pdf]

Diagnostic and Economic Evaluation of [18F]FDG PET/CT Versus MRI for Lymph-Node Staging in Nasopharyngeal Carcinoma: Implications for Individualized Upper-Neck-Only Irradiation

Supplementary Methods

PET/CT and MRI Acquisition

PET/CT was performed on integrated PET/CT systems (Discovery ST, GE Healthcare; or Biograph mCT, Siemens Healthineers) according to published guidelines for oncologic [18F]FDG PET/CT imaging.[1] Patients fasted for at least 6 hours, and blood glucose was controlled before tracer injection. The administered activity was 5.55 ± 0.74 MBq/kg (0.15 ± 0.02 mCi/kg) on the Discovery ST and 3.70 ± 0.37 MBq/kg (0.10 ± 0.01 mCi/kg) on the Biograph mCT, consistent with institutional protocols for 2D and 3D acquisitions, respectively.

Imaging was initiated 50–80 minutes after [18F]FDG injection. A low-dose CT scan for attenuation correction preceded PET emission imaging. For the Discovery ST, CT parameters included automatic tube current modulation, tube voltage 140 kV, collimation 16 × 1.25 mm, rotation time 0.8 s, and slice thickness 3.75 mm. For the Biograph mCT, parameters included tube current 80–200 mAs, tube voltage 120 kV, collimation 32 × 1.25 mm, rotation time 0.5 s, and slice thickness 3 mm. PET emission data were acquired for 3 minutes per bed position in 2D mode (Discovery ST) or 1.5–2 minutes per bed in 3D mode (Biograph mCT), with 6–8 bed positions covering the skull base to the upper thorax. PET images were reconstructed using ordered-subset expectation maximization with attenuation correction; reconstructed slice thickness was 3.25 mm (2D, 128 × 128 matrix) or 2 mm (3D, 200 × 200 matrix).

MRI was performed on 1.5-T (Signa CVi, GE Healthcare) or 3.0-T (Magnetom Tim Trio, Siemens) scanners. The scan range extended from the suprasellar cistern to the superior border of the thoracic cage. Non–contrast-enhanced sequences included fast spin–echo (FSE) T1-weighted imaging (T1WI) in axial, coronal, and sagittal planes (TR/TE 500–600/10–20 ms) and axial FSE T2-weighted imaging (T2WI; TR/TE 4000–6000/95–110 ms). After intravenous injection of gadolinium-based contrast (Gd-DTPA, 0.1 mmol/kg body weight), contrast-enhanced, fat-suppressed FSE T1WI sequences were obtained in axial, coronal, and sagittal planes.

For axial MRI sequences, the slice thickness was 5 mm with a 1 mm interslice gap; for coronal and sagittal sequences, the slice thickness was 2 mm. MRI and PET/CT were performed within 14 days (2 weeks) prior to ultrasound-guided biopsy, reflecting real-world institutional practice.

Imaging Evaluation

In Cohort A, PET/CT and MRI images were reviewed independently by experienced radiologists specialized in nuclear medicine (XZ, WF; >20 years' experience in head-and-neck cancer) and head-and-neck MRI (YH, CMX; YH reading >300 NPC cases per month for 7 years; CMX with >30 years' experience). Readers were blinded to all clinical, pathologic, and other imaging findings related to the contoured lymph nodes. Disagreements within each modality were resolved by consensus.

For both PET/CT and MRI, nodes were scored using a 5-point scale: 0, definitely benign; 1, low suspicion; 2, moderate suspicion; 3, high suspicion; 4, definitely metastatic.For statistical analysis, scores of 0–1 were classified as negative and 2–4 as positive.

Morphologic criteria for metastatic lymph nodes on MRI and CT components of PET/CT were adapted from previously established head-and-neck imaging criteria[2]. Features indicating malignancy included: (a) short-axis diameter >10 mm, (b)irregular or spiculated margin, (c) cortical inhomogeneity, (d) perifocal edema, (e) absence of fatty hilum, (f) asymmetry relative to the contralateral side, (g) contrast enhancement, and (h) blurred or indistinct nodal border.
On PET/CT, visually prominent [18F]FDG uptake exceeding background activity and not attributable to physiologic biodistribution was considered suspicious for malignancy. Semi-quantitative SUV measurements (e.g., SUVmax and lesion-to-background ratios) were available on the workstation and were used as supportive information when assigning the confidence score; however, no fixed SUV cutoff was mandated due to potential variability across scanners and reconstruction settings in this retrospective cohort. Final PET/CT assessments were made by integrating metabolic and anatomic information on the intrinsically co-registered fused PET/CT images reviewed in axial, sagittal, and coronal planes, with meticulous correlation to anatomical landmarks and nodal morphology.

Cost-Effectiveness Analysis
A decision-analytic framework consisting of an initial decision tree followed by a Markov state-transition model was constructed to compare two diagnostic strategies: initial MRI versus initial [18F]FDG PET/CT. The decision tree captured immediate diagnostic outcomes based on observed sensitivities and specificities for detecting cervical lymph-node metastases. Subsequent disease trajectories were modeled using a Markov process (Supplementary Figure S2), with health states reflecting nodal control, regional recurrence, distant metastasis, and death.
Transition probabilities and model inputs were derived from the present study's diagnostic performance, institutional data, and a targeted literature review of studies published within the previous 10 years; seminal earlier studies were also considered when necessary. (Supplementary Table S1). Particular attention was given to estimating the probability of nodal recurrence when metastatic lymph nodes were misclassified as benign and treated with upper-neck-only irradiation. A recent study reported that 19.8% of patients had metastatic lymph nodes extending below the caudal border of the cricoid cartilage (i.e., lower-neck involvement).[3] Under an upper-neck-only irradiation strategy, these lower-neck nodes would fall outside the elective target volume and may therefore carry a substantially increased risk of regional relapse if occult disease is not adequately covered. To parameterize the recurrence risk for metastatic nodes that are incorrectly staged as N0 and managed with upper-neck-only irradiation, we conservatively combined (i) the proportion of patients with lower-neck involvement (19.8%) and (ii) the inherent 3-year regional relapse risk observed under standard whole-neck irradiation (5.65%) in the same study.[3] This yielded an estimated 3-year recurrence probability of 25.44% (19.8% + 5.65%). To further assess plausibility, historical NPC series have shown substantially higher regional nodal failure when elective neck irradiation was withheld, with subsequent nodal relapse rates of approximately 30–40%.[4,5] Therefore, the above 25.44% 3-year probability was considered conservative, and we tested a wide range for this parameter in sensitivity analyses.
This parameter, along with other key inputs, was extensively tested in sensitivity analyses.
Given that 10-year overall survival in nasopharyngeal carcinoma exceeds 50% in contemporary series, the total time horizon of the Markov model was set to 10 years from

initial diagnosis. The model used a 1-year cycle length, resulting in 10 cycles. Costs were expressed in US dollars, and health outcomes in quality-adjusted life years (QALYs). A willingness-to-pay (WTP) threshold of US$100,000 per QALY was adopted as a commonly used benchmark to facilitate international comparability.

Deterministic (one-way) sensitivity analyses were conducted over 95% confidence intervals for sensitivity and specificity, as well as wide plausible ranges for the probabilities of initial nodal metastasis, distant metastasis, and nodal recurrence, to evaluate their impact on the incremental cost-effectiveness ratio (ICER). To address parameter uncertainty jointly, probabilistic sensitivity analysis was performed by assigning γ-distributions to cost parameters and β-distributions to probabilities and utilities. Monte Carlo simulation with 1,000 iterations was used to estimate the joint distribution of costs and QALYs and the probability that PET/CT would be cost-effective at the WTP threshold.

All economic analyses were conducted using dedicated decision-analysis software (TreeAge Pro 2022, Williamstown, MA, USA).

Supplementary Results

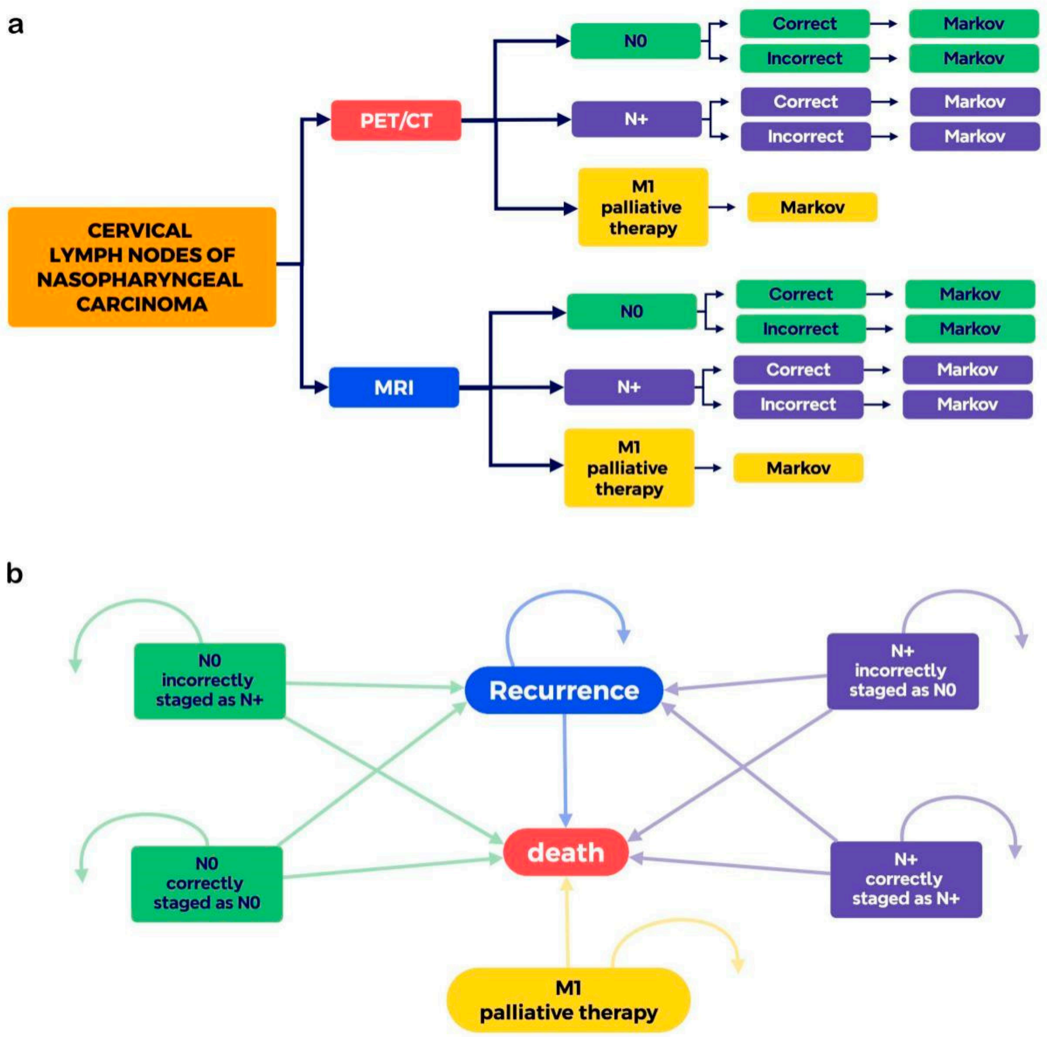

Figure S1. The economic decision tree (a) and Markov transition state model (b). MRI, magnetic resonance imaging; PET/CT, [18F] fluorodeoxyglucose positron emission tomography/computed tomography; N0, no cervical lymph node metastasis; N+, cervical lymph node metastasis

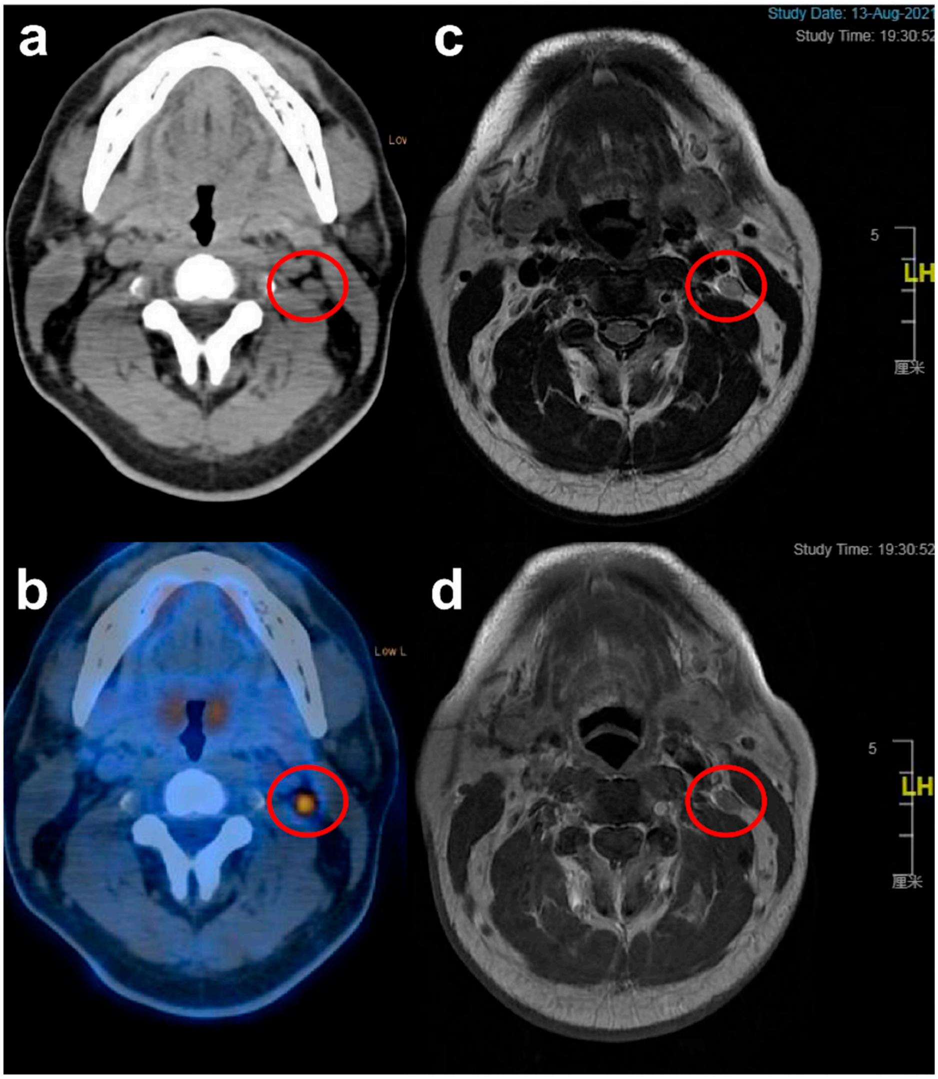

Figure S2. A pathological positive lymph node in level II of the left neck. It was correctly diagnosed by [18F] fluorodeoxyglucose positron emission tomography/computed tomography (a-b) but misdiagnosed by magnetic resonance imaging with sequences of T2-weighted imaging (c) and T1-weighted imaging with contrast (d).

Table S1. Markov model input parameters

Variable	Baseline estimate	Range studied
**Pre-test probability of cervical lymph node metastasis**	69.1%[6]	62.2%-79.6%[6–8]
**Pre-test probability of initial distant metastasis**	14.8%[9]	14.4%-20.3%[9]
**Expected age at the diagnostic procedure**	46[10]	45-55[11]
**Assumed willingness-to-pay per QALY**	100000	Assumption
**Discount rate**	3%	1%-5%
**Markov model time horizon**	10 years	
**Diagnostic test reliabilities**		
Sensitivity of MRI for cervical lymph node metastasis	92.6%	90.4%-94.8% (Cohort A)
Specificity of MRI for cervical lymph node metastasis	59.0%	50.8%-67.2% (Cohort A)
Sensitivity of PET/CT for cervical lymph node metastasis	96.0%	94.4%-97.7% (Cohort A)
Specificity of PET/CT for cervical lymph node metastasis	64.0%	56.1%-72.0% (Cohort A)
**Costs (Acute)**		
MRI (including MRI of nasopharynx and neck + chest and abdominal CT + whole body bone scan)	669	Local charge
PET/CT	1252	Local charge
Intensity-modulated radiotherapy + chemotherapy	12179	10488-21915 (Local charge)
**Costs (long-term)**		
Follow-up	255	
Palliative therapy	28986	
Recurrence	11014	
**Utilities**		
Tumor-free after upper-neck irradiation	0.836	0.636-1.000[11]
Tumor-free after whole-neck irradiation	0.760	0.610-0.910[11]
Recurrence	0.4	
Palliative therapy	0.35	0.28-0.42
Death	0	Assumption
**Transition probabilities**		
Recurrence rate of negative cervical lymph nodes if correctly staged as N0 and received upper-neck irradiation	2.68%	3-year[11]
Recurrence rate of negative cervical lymph nodes if incorrectly staged as N+ and received whole-neck irradiation	3.15%	3-year[11]
Recurrence rate of positive cervical lymph nodes if incorrectly staged as N0 and received upper-neck irradiation	25.44%	3-year[3]
Recurrence rate of positive cervical lymph nodes if correctly staged as N+ and received whole-neck irradiation	5.65%	3-year[3]
Death for the metastasized diseased (initially and after recurrence)	17%/year	SEER medicare dart
Death risk by palliative therapy	53.97%	2-year
Death without recurrence depending on age	3%	

Abbreviations: MRI, magnetic resonance imaging; PET/CT, [^{18}F] fluorodeoxyglucose positron emission tomography/computed tomography; QALY, quality-adjusted life year

Table S2. Diagnostic performance of PET/CT vs MRI at node-level (cutoff ≥3)

Metric	PET/CT	MRI	P value
ACC (95% CI)	89.3%(86.8%–91.5%)	85.3% (83.4%–87.9%)	-

SEN (95% CI)	92.1% (89.5%–92.4%)	89.7% (86.9%–92.1%)	0.080
SPE (95% CI)	78.4% (70.6%–84.9%)	67.6% (59.2%–75.3%)	0.016
PPV (95% CI)	94.5% (92.2%–96.2%)	91.7% (89.1%–93.9%)	0.011
NPV (95% CI)	71.2% (63.4%–78.3%)	62.3% (54.0%–70.0%)	0.011
AUC	0.864	0.841	0.298

Abbreviations: ACC: Accuracy; SEN: Sensitivity; SPE: Specificity; PPV: Positive Predictive Value; NPV: Negative Predictive Value; AUC: area under the curve

References

1. Delbeke D.; Coleman R.E.; Guiberteau M.J.; Brown M.L.; Royal H.D.; Siegel B.A.; Townsend D.W.; Berland L.L.; Parker J.A.; Hubner K.; et al. Procedure guideline for tumor imaging with 18F-FDG PET/CT 1.0. *J Nucl Med* **2006**, *47*, 885–895.

2. van den Brekel M.W.; Stel H.V.; Castelijns J.A.; Nauta J.J.; van der Waal I.; Valk J.; Meyer C.J.; Snow G.B. Cervical lymph node metastasis: assessment of radiologic criteria. *Radiology* **1990**, *177,* 379–384, doi:10.1148/radiology.177.2.2217772.

3. Mao, Y.-P.; Wang, S.-X.; Gao, T.-S.; Zhang, N.; Liang, X.-Y.; Xie, F.-Y.; Zhang, Y.; Zhou, G.-Q.; Guo, R.; Luo, W.-J.; et al. Medial Retropharyngeal Nodal Region Sparing Radiotherapy versus Standard Radiotherapy in Patients with Nasopharyngeal Carcinoma: Open Label, Non-Inferiority, Multicentre, Randomised, Phase 3 Trial. *BMJ* **2023**, *380,* e072133, doi:10.1136/bmj-2022-072133.

4. Lee A.W.; Sham J.S.; Poon Y.F.; Ho J.H. Treatment of stage I nasopharyngeal carcinoma: analysis of the patterns of relapse and the results of withholding elective neck irradiation. *Int J Radiat Oncol Biol Phys* **1989**, *17,*

1183–1190, doi:10.1016/0360-3016(89)90524-5.

5. Lee A.W.; Poon Y.F.; Foo W.; Law S.C.; Cheung F.K.; Chan D.K.; Tung S.Y.; Thaw M.; Ho J.H. Retrospective analysis of 5037 patients with nasopharyngeal carcinoma treated during 1976-1985: overall survival and patterns of failure. *Int J Radiat Oncol Biol Phys* **1992**, *23*, 261–270, doi:10.1016/0360-3016(92)90740-9.

6. OuYang P.-Y.; Su Z.; Ma X.-H.; Mao Y.-P.; Liu M.-Z.; Xie F.-Y. Comparison of TNM staging systems for nasopharyngeal carcinoma, and proposal of a new staging system. *Br J Cancer* **2013**, *109*, 2987–2997, doi:10.1038/bjc.2013.659.

7. Chin O.; Yu E.; O'Sullivan B.; Su J.; Tellier A.; Siu L.; Waldron J.; Kim J.; Hansen A.; Hope A.; et al. Prognostic importance of radiologic extranodal extension in nasopharyngeal carcinoma treated in a Canadian cohort. *Radiother Oncol* **2021**, *165*, 94–102, doi:10.1016/j.radonc.2021.10.018.

8. Ma H.; Liang S.; Cui C.; Zhang Y.; Xie F.; Zhou J.; Dong A.; Chen M.; Xie C.; Li H.; et al. Prognostic significance of quantitative metastatic lymph node burden on magnetic resonance imaging in nasopharyngeal carcinoma: A retrospective study of 1224 patients from two centers. *Radiother Oncol* **2020**, *151*, 40–46, doi:10.1016/j.radonc.2020.07.023.

9. Tang L.-Q.; Chen Q.-Y.; Fan W.; Liu H.; Zhang L.; Guo L.; Luo D.-H.; Huang P.-Y.; Zhang X.; Lin X.-P.; et al. Prospective study of tailoring whole-body dual-modality [18F]fluorodeoxyglucose positron emission tomography/computed tomography with plasma Epstein-Barr virus DNA for

detecting distant metastasis in endemic nasopharyngeal carcinoma at initial staging. *J Clin Oncol* **2013**, *31*, 2861–2869, doi:10.1200/JCO.2012.46.0816.

10. Sun X.-S.; Liu S.-L.; Luo M.-J.; Li X.-Y.; Chen Q.-Y.; Guo S.-S.; Wen Y.-F.; Liu L.-T.; Xie H.-J.; Tang Q.-N.; et al. The Association Between the Development of Radiation Therapy, Image Technology, and Chemotherapy, and the Survival of Patients With Nasopharyngeal Carcinoma: A Cohort Study From 1990 to 2012. *Int J Radiat Oncol Biol Phys* **2019**, *105*, 581–590, doi:10.1016/j.ijrobp.2019.06.2549.

11. Tang, L.-L.; Huang, C.-L.; Zhang, N.; Jiang, W.; Wu, Y.-S.; Huang, S.H.; Mao, Y.-P.; Liu, Q.; Li, J.-B.; Liang, S.-Q.; et al. Elective Upper-Neck versus Whole-Neck Irradiation of the Uninvolved Neck in Patients with Nasopharyngeal Carcinoma: An Open-Label, Non-Inferiority, Multicentre, Randomised Phase 3 Trial. *Lancet Oncol* **2022**, *23*, 479–490, doi:10.1016/S1470-2045(22)00058-4.
